# Multimodality cardiovascular imaging in arrhythmic mitral valve prolapse: a state-of-the-art review from structural assessment to myocardial tissue characterization

**DOI:** 10.3389/fcvm.2026.1854022

**Published:** 2026-06-15

**Authors:** Giovanni Taverna, Kristian Galanti, Annagrazia Cecere, Francesca Graziano, Giancarlo Trimarchi, Roberta Antonazzo Panico, Antonella Cecchetto, Monica De Gaspari, Manuel Signorini, Aldo Carnevale, Giorgio De Conti, Raffaella Motta, Alberto Cipriani, Leonardo Calò, Fabrizio Ricci, Cristina Basso, C. Anwar A. Chahal, Martina Perazzolo Marra

**Affiliations:** 1 PhD Program in Translation Specialistic Medicine “G.B. Morgagni”, Curriculum “Cardiovascular Sciences”, University of Padua, Padua, Italy; 2 Department of Translational Medicine, Section of Radiology, University of Ferrara, Ferrara, Italy; 3 Department of Cardiology, Policlinico Casilino, Rome, Italy; 4 Center for Inherited Cardiovascular Diseases, Genomic and Precision Medicine, WellSpan Health, York, PA, United States; 5 Department of Cardiac, Thoracic, and Vascular Sciences and Public Health, University of Padua, Padua, Italy; 6 Health Science Interdisciplinary Center, Scuola Superiore Sant'Anna, Pisa, Italy; 7 Fondazione Toscana G. Monasterio, Ospedale del Cuore “G. Pasquinucci”, Massa, Italy; 8 Cardiology Unit, Città di Lecce Hospital, Lecce, Italy; 9 Cardiovascular Pathology Unit, Azienda Ospedale-Università Padova, Padua, Italy; 10 Radiology Department, S. Maria Della Misericordia Hospital, ULSS 5 Polesana, Rovigo, Italy; 11 Radiology Unit, University Hospital of Padua, Padua, Italy; 12 Department of Movement, Human and Health Sciences, University of Rome “Foro Italico”, Rome, Italy; 13 Department of Neuroscience, Imaging and Clinical Sciences, “G. D’Annunzio” University of Chieti-Pescara, Chieti, Italy; 14 Institute for Advanced Biomedical Technologies, “G. D’Annunzio” University of Chieti-Pescara, Chieti, Italy; 15 Department of Clinical Sciences, Lund University, Malmö, Sweden; 16 University Cardiology Division, Heart Department, SS Annunziata Hospital, ASL 02 Abruzzo, Chieti, Italy; 17 Department of Cardiology, WellSpan Health, York, PA, United States; 18 William Harvey Research Institute, NIHR Barts Biomedical Research Centre, Queen Mary University London, London, United Kingdom

**Keywords:** arrhythmic mitral valve prolapse, cardiac magnetic resonance, mitral valve prolapse, multimodality imaging, myocardial injury, positron - emission tomography, precision medicine, ventricular arrhythmias

## Abstract

Mitral valve prolapse (MVP) is a prevalent and traditionally benign heart valve disease; however, mounting evidence identifies a subset of patients at risk for malignant ventricular arrhythmias and sudden cardiac death. This narrative review critically examines the role of multimodality imaging in arrhythmic MVP, with an integrative overview of transthoracic echocardiography, cardiac magnetic resonance (CMR), computed tomography, and positron emission tomography. Particular attention is given to the complementary contribution of these modalities to structural, functional, and myocardial tissue characterization, while acknowledging their different levels of evidence and current clinical applicability. The specific added value of this review lies in organizing these imaging findings within a pathophysiological framework that links valve morphology and annular biomechanics to myocardial injury, diffuse fibrotic remodeling, focal replacement fibrosis, and arrhythmic vulnerability, rather than presenting each modality as an isolated diagnostic tool. Advanced CMR techniques, including native T1 and T2 mapping and extracellular volume quantification, are discussed as promising tools for detecting diffuse fibrosis and myocardial tissue characterization that may contribute to early myocardial remodeling. Genetic susceptibility is also considered as a potential modifier of phenotypic heterogeneity rather than as a routine component of current risk stratification. Overall, the reviewed literature supports an evolving framework for phenotypic characterization and mechanistic interpretation in arrhythmic MVP, while highlighting the need for further standardization, prospective validation, and cautious integration of advanced imaging biomarkers into clinical pathways.

## Graphical Abstract

**Figure:**
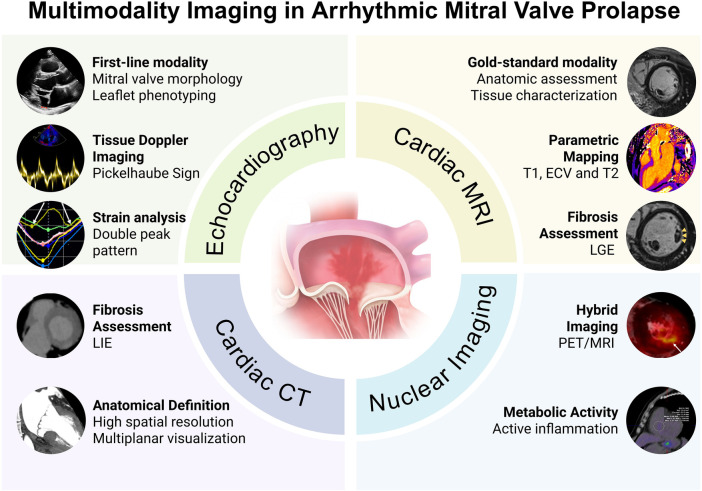
Multimodality imaging markers across the spectrum of arrhythmic mitral valve prolapse from structural assessment to myocardial tissue characterization. Echocardiography, cardiac magnetic resonance, cardiac computed tomography, and hybrid PET/MRI features collectively support phenotypic characterization and arrhythmic risk stratification.

## Introduction

Mitral valve prolapse (MVP) represents one of the most common heart valve disorders worldwide, affecting approximately 2%–3% of the general population (1). It is defined as a systolic displacement of one or both mitral leaflets by ≥ 2 mm above the plane of the mitral annulus in the parasagittal view (2). Two principal morphological patterns are recognized: (I) myxomatous MVP, exemplified by Barlow's disease, in which redundant leaflet tissue is accompanied by chordal thickening and/or elongation; (II) fibroelastic deficiency, characterized by leaflet thinning and chordal attenuation (3). In contrast, mitral annular disjunction (MAD) refers to the atrial displacement of the posterior mitral leaflet hinge point from the underlying left ventricular myocardium (4). Recent data indicate that this displacement may occur only in systole (pseudo-MAD), or both in systole and diastole (true MAD), highlighting MAD as a dynamic, multiphase abnormality rather than a purely systolic phenomenon (5). Although traditionally regarded as a largely benign entity, increasing evidence indicates that a subset of individuals with MVP may develop malignant ventricular arrhythmias (VAs), occasionally culminating in sudden cardiac death (SCD). This heterogeneity has prompted the recognition of an arrhythmic form of MVP, called arrhythmic mitral valve prolapse (aMVP), characterized by complex premature ventricular contractions (PVC), non-sustained (NSVT) or sustained ventricular tachycardia (SVT), or out-of-hospital cardiac arrest in individuals without severe mitral regurgitation or overt cardiomyopathy (6, 7). According to the 2022 EHRA Expert Consensus Statement on aMVP and MAD, the diagnosis of aMVP requires the presence of at least 5% PVC daily burden or complex VAs, such as NSVT, VT or ventricular fibrillation, with or without the presence of MAD (8). Over the past decade, this phenotype has emerged as an important clinical and research focus, with efforts directed toward understanding its mechanisms, identifying markers of risk, and improving the detection of vulnerable patients. Multiple epidemiological and pathological studies have shown that MVP is overrepresented among individuals with unexplained SCD, particularly in young women and athletes, suggesting that the arrhythmogenic burden of the condition may be substantially higher than previously appreciated (9). Post-mortem investigations have repeatedly demonstrated localized fibrosis in the basal inferolateral left ventricular wall and papillary muscles (PMs) in victims of MVP-related SCD, reinforcing the hypothesis that structural myocardial remodeling underlies at least part of the arrhythmogenic substrate (6, 10). Yet, structural abnormalities alone do not fully explain the phenotype. Arrhythmias occur in patients with minimal leaflet redundancy, preserved LV function, and absent severe mitral regurgitation. Conversely, many individuals with pronounced myxomatous disease exhibit no arrhythmias. This inconsistency highlights a crucial knowledge gap: anatomy and valve mechanics alone cannot entirely account for the arrhythmogenic risk, and additional determinants, such as electrophysiologic, metabolic, inflammatory, and genetic, are likely involved. MAD has received considerable attention in the last decades (11). Several studies have shown a strong association between MAD and complex VAs, while others have questioned its prognostic value in population-based cohorts (12). Dynamic features of the annulus, as systolic “curling” exaggerated saddle-shape changes, and altered mitral-ventricular coupling, have been proposed as mechanisms precipitating papillary-muscle traction and mechanical stress on the inferolateral myocardium (10, 13). Nonetheless, MAD is not uniformly present among patients with severe arrhythmias, nor is it sufficient to generate a malignant phenotype in isolation (14). These observations collectively suggest that arrhythmogenesis in MVP is multifactorial, involving a complex interplay between mechanical forces, myocardial vulnerability, electrophysiological triggers, and possibly genetic predisposition.

Although several reviews have addressed arrhythmic mitral valve prolapse and isolated imaging features, a focused synthesis of how multimodality imaging may characterize the transition from structural valvular abnormalities to myocardial tissue remodeling remains timely. The specific added value of this review is the proposal of an integrated imaging framework in which echocardiography primarily defines valve morphology, mitral regurgitation, annular dynamics, and biomechanical stress; CMR links these structural abnormalities to ventricular remodeling, focal scar, and diffuse tissue characterization; CT is positioned as a selective anatomical tool rather than a routine tissue-characterization modality; and PET/MRI is discussed as an investigational technique for metabolic or inflammatory myocardial activity. This structure is intended to move beyond a modality-by-modality catalogue and to interpret imaging findings along a pathophysiological continuum from mechanical stress to myocardial injury, fibrotic remodeling, and arrhythmic vulnerability, while explicitly distinguishing clinically established markers from promising but still non-validated biomarkers. The aim of this narrative review is therefore to provide a critical multimodality overview of arrhythmogenic MVP, with particular emphasis on phenotypic characterization, tissue-level assessment, evidence maturity, and clinically oriented risk interpretation (graphical abstract).

## Methods of literature search and study selection

This article was conceived as a narrative review. A focused search of PubMed/MEDLINE, Scopus, and Google Scholar was performed for studies published up to January 2026 on arrhythmic mitral valve prolapse, mitral annular disjunction, ventricular arrhythmias, and multimodality imaging, with particular attention to echocardiography, cardiac magnetic resonance, cardiac CT, PET/MRI, fibrosis, edema, and inflammation. Priority was given to original studies, expert consensus documents, and recent clinically relevant reviews. Reference lists of selected papers were also manually screened. No formal systematic review protocol or meta-analysis was applied.

## Epidemiology

MVP is the most common valvular abnormality, affecting approximately 2%–3% of the general population (15, 16). Although MVP without significant mitral regurgitation is generally regarded as a benign condition, a distinct subset of patients exhibits a markedly increased susceptibility to VAs and SCD. The estimated incidence of SCD in unselected MVP cohorts is below 1% (17, 18), with community-based data suggesting an event rate of roughly 0.14 per 100 patient-years (18). However, given the high prevalence of MVP, even a relatively low event rate may remain clinically relevant at the population level. Notably, MVP has been identified as the most likely underlying cause in 11.7% of unexplained SCD cases, and a recent meta-analysis reported a 1.9% prevalence of MVP among all SCD victims, further underscoring its relevance within arrhythmic risk stratification (19).

## Historical background

The notion that MVP might predispose to malignant VAs emerged nearly half a century ago. In the mid-1970s, several clinical observations challenged the prevailing view of MVP as a benign entity. One of the earliest pivotal reports was published by Jeresaty in 1976, describing cases of sudden death in the context of the “mitral valve prolapse-click syndrome” (20). These early descriptions linked auscultatory features (e.g., systolic clicks and late systolic murmurs) to unexpected fatal arrhythmic outcomes, suggesting that a subgroup of MVP patients might harbor an intrinsic electrical vulnerability. Concurrently, other investigators refined the characterization of the electrical disturbances associated with MVP. De Maria and colleagues reported on the prevalence, nature, and frequency of VAs in the MVP syndrome, identifying a spectrum ranging from isolated to complex and repetitive PVCs (21). These observations hinted at a possible mechanistic relationship between leaflet prolapse, myocardial traction, and arrhythmogenesis. A series of influential case descriptions further expanded the field. In the British Heart Journal (1976), Campbell, et al. reported VAs in the “balloon deformity” of the mitral valve, identifying a possible “high-risk group.” Their integration of echocardiographic findings, such as particularly exaggerated posterior leaflet displacement, with concomitant malignant arrhythmias represented an early attempt to define morphological predictors of electrical instability. The inclusion of simultaneous M-mode recordings and electrocardiograms in these early works underscored the concept that abnormal leaflet–myocardial interaction could translate into arrhythmic triggers (22). During this period, several authors also proposed that MVP could represent a “cardiomyopathic state” rather than an isolated valvular abnormality. Gulotta et al. explored whether the “systolic click-murmur syndrome” might reflect a broader myocardial disorder (23). Crawford and O’Rourke subsequently echoed this perspective, arguing that MVP may share features with primary cardiomyopathies, particularly in cases with diffuse leaflet thickening, PMs anomalies, or repolarization abnormalities (24). Although these early insights were conceptually ahead of their time, the field lacked standardized diagnostic criteria, multimodal imaging, and systematic longitudinal follow-up. As a result, the arrhythmogenic implications of MVP remained controversial for decades. The modern era, enabled by cardiac magnetic resonance (CMR), advanced echocardiography, and large observational cohorts, has provided the tools necessary to validate and refine these historical hypotheses. The 2022 EHRA Expert Consensus Statement on Arrhythmic MVP and MAD synthesized decades of evidence, formally defining the “*arrhythmic MVP complex”*, delineating phenotypic risk markers, and establishing a structured framework for diagnosis, imaging, and arrhythmic surveillance (8). This landmark document represents the culmination of the trajectory initiated by the early case series of the 1970s, transforming isolated clinical observations into a robust, multidimensional model of disease.

## The role of multimodality imaging in arrhythmic mitral valve prolapse

For clarity, the following sections are organized according to the main conceptual contribution of each modality within the proposed framework: structural and biomechanical characterization, myocardial tissue characterization, and emerging metabolic or inflammatory assessment. This structure is intended to highlight not only the complementarity of the different imaging techniques, but also the different levels of maturity and current clinical applicability of the imaging markers discussed.

Contemporary cardiovascular imaging modalities, including transthoracic echocardiography (TTE), computed tomography (CT), CMR, and hybrid positron emission tomography/magnetic resonance imaging (PET/MRI), provide complementary and non-redundant information that is key to comprehensive phenotyping and risk stratification in aMVP (25). An integrated, multimodality approach enables the identification and characterization of key structural and functional features, including MAD, annular curling, bileaflet prolapse, leaflet redundancy and thickening, as well as focal and diffuse myocardial fibrosis, which together constitute the arrhythmogenic substrate. Each imaging modality contributes distinct yet synergistic insights: echocardiography allows dynamic assessment of valve morphology and mechanics; CT offers high-resolution delineation of mitral annular anatomy and adjacent structures and CMR provides accurate functional quantification coupled with advanced myocardial tissue characterization. Also, PET/MRI enables evaluation of metabolic and inflammatory myocardial activity relevant to arrhythmogenesis. Collectively, these techniques provide complementary information for phenotypic characterization of arrhythmogenic MVP and may help refine mechanistic interpretation and risk-oriented evaluation, although their current levels of validation and clinical applicability remain heterogeneous across modalities.

### Echocardiography

**Figure 1:**
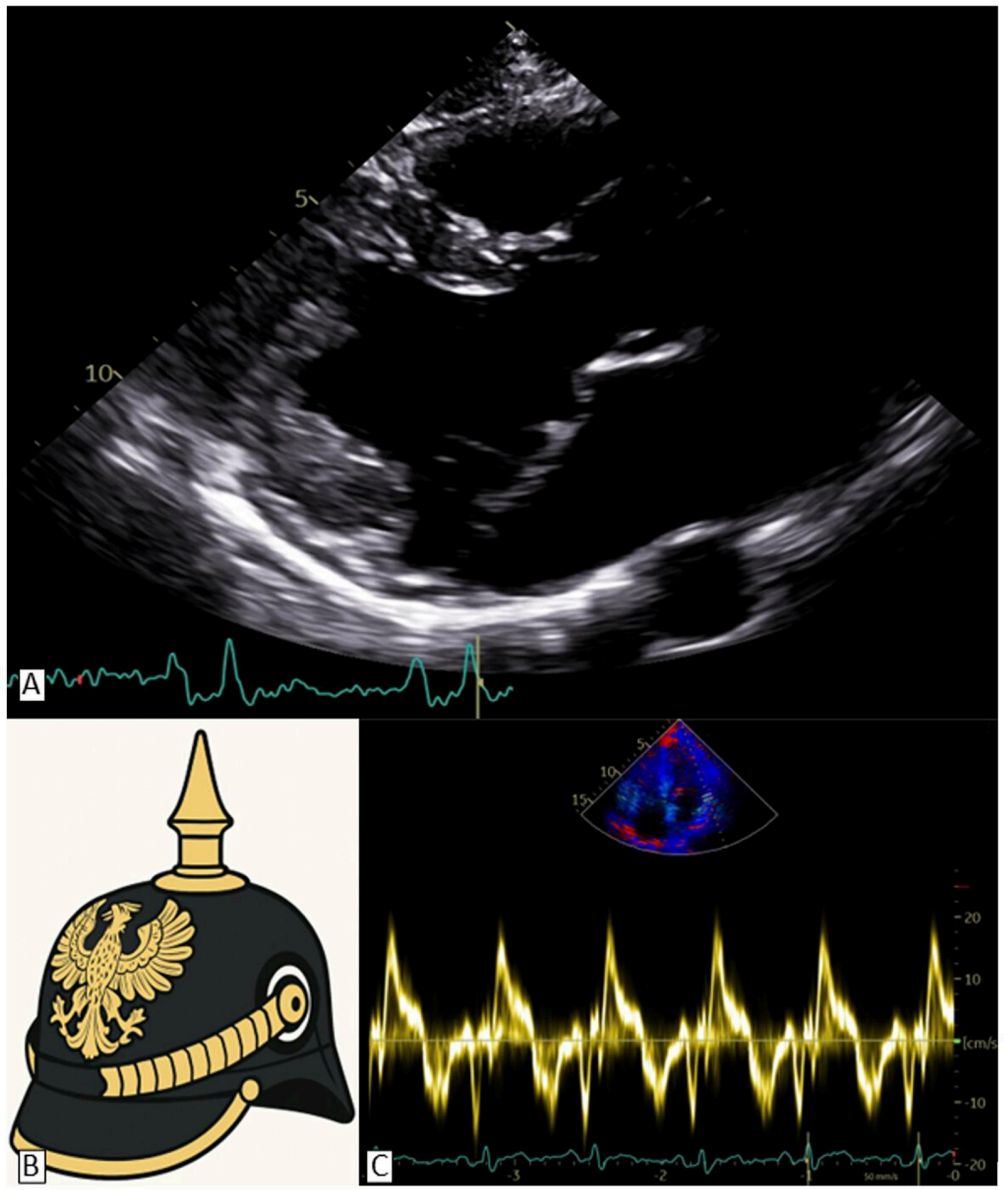
Echocardiographic and Doppler features of mitral annular systolic motion. **(A)** Parasternal long axis view showing bileaflet mitral valve prolapse with associated annular disjunction. **(B)** Illustration of the “Pickelhaube sign,” named after the characteristic sharp velocity spikes resembling the Prussian spiked helmet. **(C)** Spectral Doppler tracing demonstrating elevated systolic mitral annular velocities consistent with increased mechanical stress.

**Figure 2:**
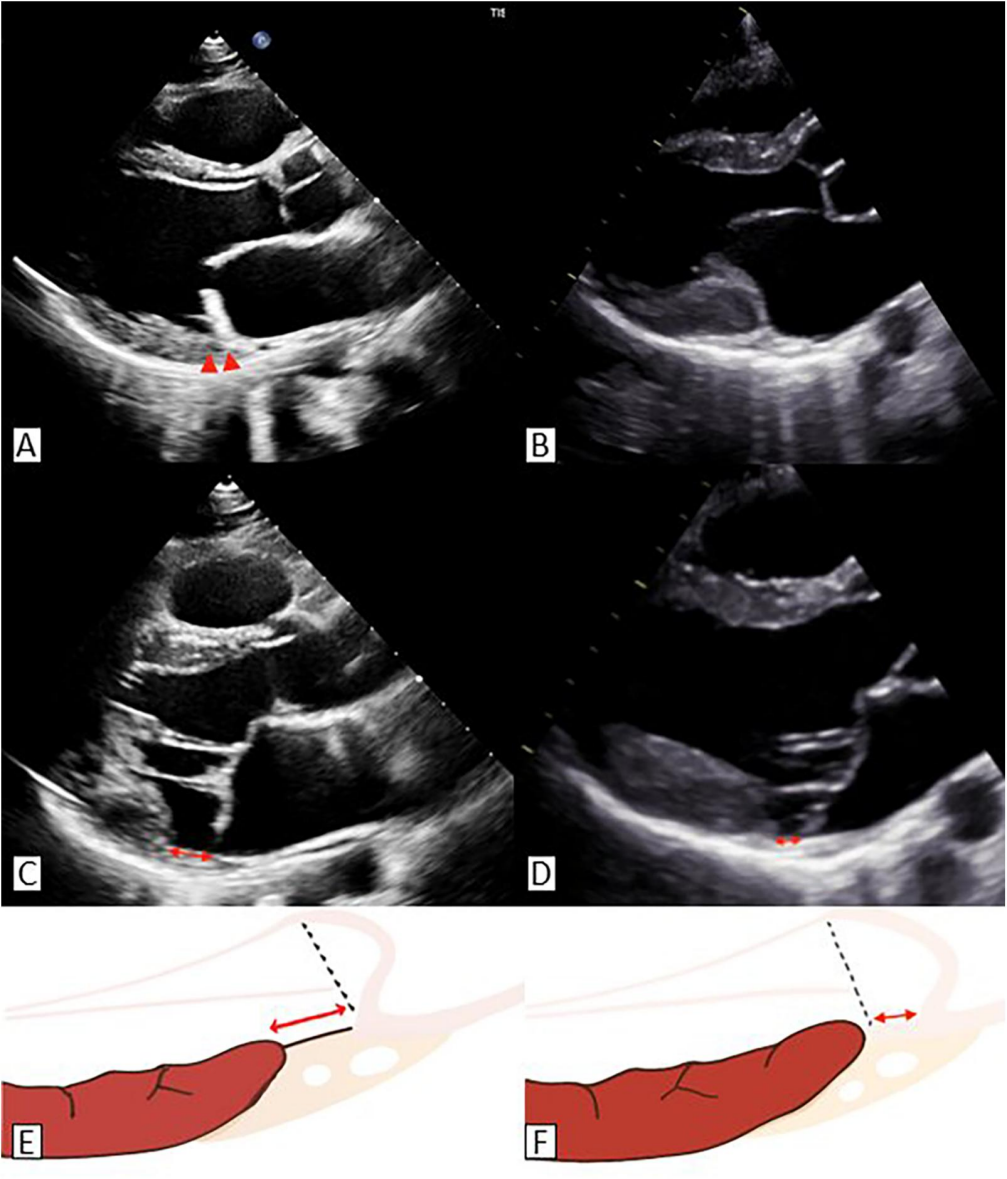
Transthoracic echocardiography. Parasternal long axis views demonstrating true mitral annular disjunction (MAD) and pseudo-MAD **(A,B)**. True MAD is defined as separation of the posterior mitral leaflet hinge point from the left ventricular myocardium, visible throughout diastole **(A)** and systole **(C)**. Pseudo-MAD **(B)** shows displacement limited to systole **(D)**. Red double arrows indicate the measured separation distance; the arrowheads in **(A)** highlight the leaflet hinge point separation during diastole in true MAD. Corresponding schematic illustrations depict true MAD **(E)** and pseudo-MAD **(F)**, emphasizing their dynamic differences using red double arrows. MAD, mitral annular disjunction.

Transthoracic echocardiography remains the first-line imaging modality for the diagnosis and initial phenotyping of MVP, allowing real-time assessment of mitral valve morphology, leaflet motion, and valvular function. It reliably identifies key valvular abnormalities, including leaflet prolapse, billowing, and mitral regurgitation. Myxomatous degeneration is predominantly driven by proteoglycan accumulation within the leaflet tissue, leading to leaflet thickening, redundancy, chordal elongation, and annular dilatation. Echocardiographic features most consistently associated with arrhythmic MVP include bileaflet prolapse, excessive leaflet thickening, MAD, and dynamic markers of increased mechanical stress, such as the Pickelhaube sign, defined as a systolic spike in mitral annular velocity with a lateral S’ ≥ 16 cm/s (Figure 1) (26). MAD, initially described by Bharati et al. (27) and later systematically characterized by Hutchins (28), refers to a spatial separation between the posterior mitral annulus and the basal left ventricular myocardium, which can be readily visualized in long-axis views using both two- and three-dimensional echocardiography. In contrast, pseudo-MAD represents an apparent annular separation caused by systolic apposition of the posterior mitral leaflet against the atrial wall, without true detachment from the ventricular myocardium (Figure 2) (29). The distinction between true-MAD and pseudo-MAD may have potential mechanistic relevance (5, 29). True-MAD, by persisting throughout both systole and diastole, may reflect a more fixed abnormal annular–myocardial relationship and a persistent alteration of mitral–ventricular coupling. Pseudo-MAD, conversely, should not be regarded simply as an irrelevant artefact: although the apparent separation is restricted to systole and is related to leaflet apposition rather than true anatomical detachment, it may still identify a highly dynamic myxomatous phenotype characterized by exaggerated leaflet motion, annular hypermobility, curling, papillary muscle traction, and abnormal basal myocardial deformation (29, 31). Adjacent myocardial abnormalities, including annular “curling,” defined as a brisk systolic upward displacement of the posterior mitral annulus, can be evaluated through frame-by-frame analysis to better localize leaflet hinge points. This abnormal annular motion was originally reported in 1976 by Gilbert et al. (30), who described a characteristic systolic curling of the posterior mitral annulus in patients with MVP. Perazzolo Marra et al. (14) later showed a close relationship between MAD and annular curling, identifying this exaggerated systolic displacement as a marker of increased annular hypermobility. More recent studies have shown a strong association between MAD, abnormal myocardial strain patterns, and an increased risk of malignant VAs in MVP (31). However, direct prognostic comparisons between true-MAD and pseudo-MAD remain limited. Available data suggest that the arrhythmic significance of MAD is unlikely to depend on its binary presence alone, but rather on the interaction among timing of the disjunction, inferolateral location, longitudinal extent, annular curling, myocardial deformation abnormalities, and the presence of myocardial fibrosis (14, 31, 32). Therefore, the true-MAD/pseudo-MAD distinction should currently be interpreted as an emerging phenotyping tool and a hypothesis-generating marker, rather than as a validated stand-alone risk-stratification criterion. Building on these structural and dynamic observations, speckle-tracking echocardiography (STE) has emerged as a valuable adjunctive tool for refining arrhythmic risk assessment by enabling quantitative analysis of myocardial deformation (32). STE has revealed subtle but reproducible abnormalities in basal inferolateral left ventricular strain, reflecting myocardial stretch and early fibrotic remodeling that may serve as substrates for VAs (32). Patients with MVP frequently exhibit pronounced strain impairment in basal ventricular segments, abnormalities that have been shown to partially reverse following mitral valve surgery, likely in relation to reduced annular dimensions and mechanical stress. The repetitive mechanical forces imposed by leaflet prolapse promote segmental myocardial lengthening and attenuated deformation within the basal myocardium, providing mechanistic insight into prolapse-related myocardial injury. In parallel, increased mechanical dispersion (MD), a STE-derived marker of heterogeneous ventricular contraction, has been consistently associated with aMVP. In the study by Ermakov et al. (33), patients with aMVP exhibited significantly higher MD values compared with non-arrhythmic MVP patients, an association that remained robust after adjustment for demographic variables, left ventricular ejection fraction, and global strain, supporting MD as an independent predictor of arrhythmic risk. Moreover, a characteristic double-peak strain pattern, with pre-telesystolic and post-telesystolic components in the inferolateral wall, has been described in patients with myocardial fibrosis and shows a strong correlation with ventricular arrhythmic events (34). STE may represent a useful adjunctive tool in selected patients when conventional echocardiographic findings do not fully explain the suspected arrhythmic phenotype or when further refinement of myocardial mechanical abnormalities is clinically warranted. Overall, echocardiography remains the cornerstone of first-line assessment in MVP, as it provides the most accessible and clinically established framework for identifying valvular morphology, regurgitation severity, annular abnormalities, and dynamic mechanical markers associated with arrhythmic phenotype. However, its ability to characterize myocardial tissue-level changes remains limited, thereby supporting the complementary role of advanced cross-sectional imaging in selected patients.

### Computed tomography

**Figure 3:**
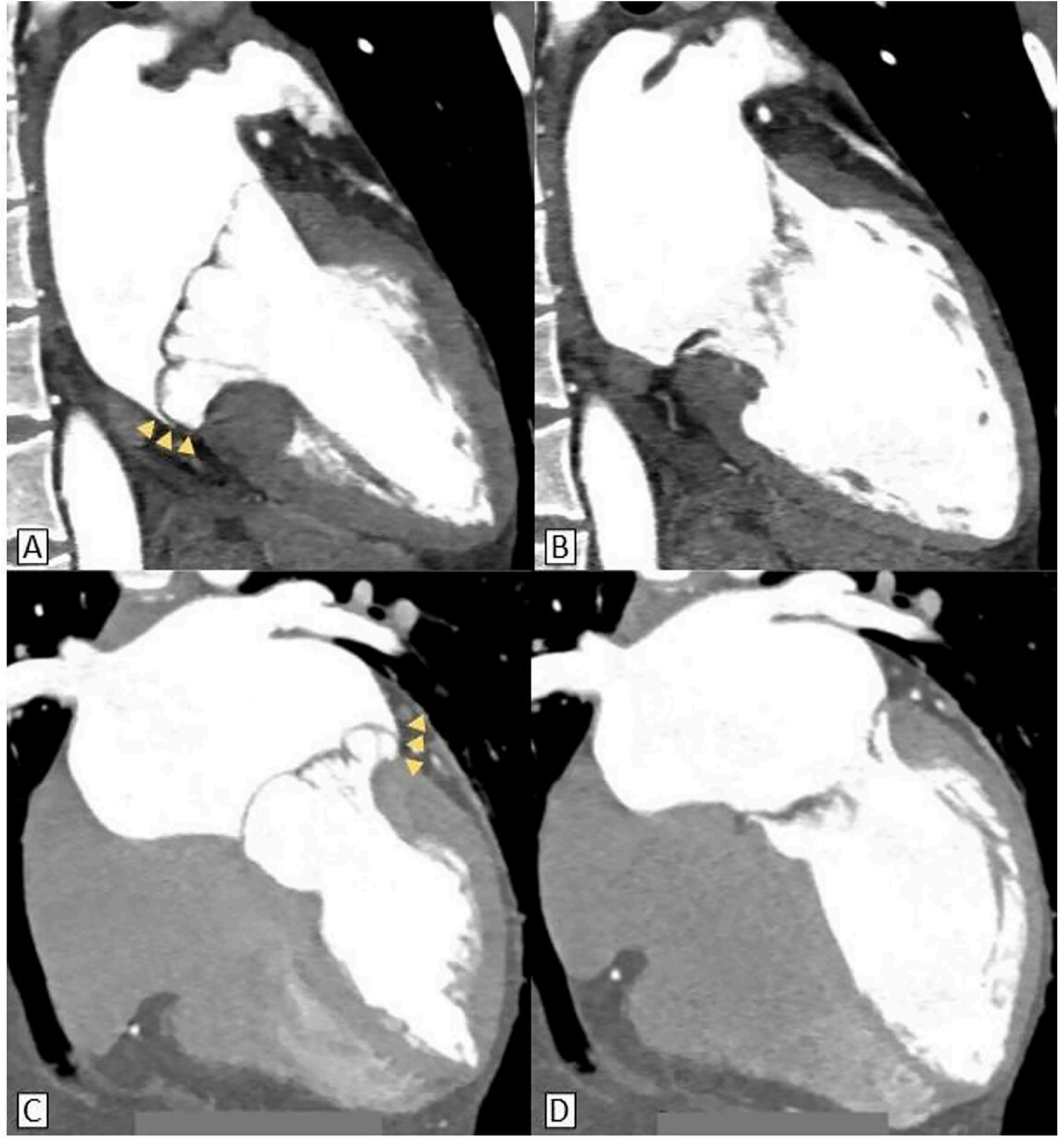
Cardiac computed tomography (CT). The figures show myxomatous mitral leaflets in four- and two-chamber views during systole and diastole **(A–D)**. CT offers high spatial resolution and multiplanar visualization of the mitral valve apparatus, revealing systolic disjunction **(A,C)**, characterized by separation of the posterior mitral leaflet hinge point from the underlying left ventricular myocardium.

Computed Tomography complements echocardiography by providing high spatial resolution and multiplanar visualization of the mitral valve apparatus, the mitral annulus, and adjacent cardiac structures. Its superior anatomical definition of mitral annular morphology and precise assessment of the extent of MAD effectively overcome several limitations related to echocardiographic acoustic windows. While CT demonstrates excellent specificity for the detection of MVP (approximately 95%), its sensitivity remains comparatively lower (around 80%) than that of echocardiography (approximately 87%), particularly in the identification of bileaflet prolapse (35). Nevertheless, CT allows reliable differentiation between true MAD, reflecting a pathological annular-myocardial separation, and pseudo-MAD, an apparent systolic phenomenon without true myocardial detachment (Figure 3). In this context, ECG-gated multiphase CT may help clarify whether annular separation persists throughout the cardiac cycle or is confined to systole, particularly when echocardiographic findings are inconclusive (35, 36). This distinction may be useful for anatomical phenotyping and procedural planning, although its incremental prognostic value for arrhythmic risk remains to be established (36). This distinction is of relevance for accurate anatomical characterization and has important implications for surgical or interventional planning in patients with complex mitral valve anatomy or significant mitral regurgitation (36). It is important to emphasize that in routine clinical practice, cardiac CT is seldom utilized as a first- or second-line imaging modality in the assessment of aMVP. Instead, its use is generally reserved for specific scenarios: (I) pre-procedural planning for mitral valve surgery or transcatheter interventions when echocardiographic windows are inadequate; (II) exclusion of obstructive coronary artery disease in patients who cannot undergo CMR; and (III) resolution of discordant echocardiographic findings when high-resolution annular imaging is required. Therefore, CT should be considered primarily a complementary tool rather than a co-equal alternative to CMR in the aMVP diagnostic pathway to avoid misinterpretation in clinical and educational settings. Although limited by ionizing radiation exposure and the need for iodinated contrast, CT holds an established role when echocardiographic results are inconclusive or conflicting. Beyond structural delineation, the detailed anatomic information provided by CT contributes to a more refined understanding of the mechanical forces acting on the mitral leaflets and annulus, which are central to the development of the arrhythmogenic substrate in aMVP. Traditionally confined to anatomical assessment due to concerns over radiation exposure, cardiac CT is now expanding with recent advances in myocardial tissue characterization, including late iodine enhancement (LIE). Similar to late gadolinium enhancement in CMR, LIE may detect delayed iodine retention in fibrotic or injured myocardial tissue; however, its use in this context is still highly preliminary and experimental. While a recent study by Taverna et al. demonstrated the technical feasibility of LIE in cardiac CT for myocardial injury detection (37), broader evidence supporting this technique is still limited (38). Additional studies and systematic reviews have begun to assess the diagnostic accuracy of LIE and extracellular volume (ECV) quantification on cardiac CT, showing promising correlations with CMR findings in fibrosis detection but highlighting the need for further validation in diverse clinical settings (39–41). Given the emerging nature of this methodology, LIE should be interpreted with caution and currently cannot replace CMR for myocardial tissue characterization in aMVP. More robust clinical data are necessary to establish its utility and prognostic value. Consequently, current clinical use of cardiac CT remains primarily focused on detailed anatomical mapping (mitral annulus, leaflets, and adjacent structures) especially when echocardiography is inconclusive or CMR is not feasible, but it is not yet established as a tool for myocardial tissue-based risk stratification in aMVP.

### Cardiac magnetic resonance

**Figure 4:**
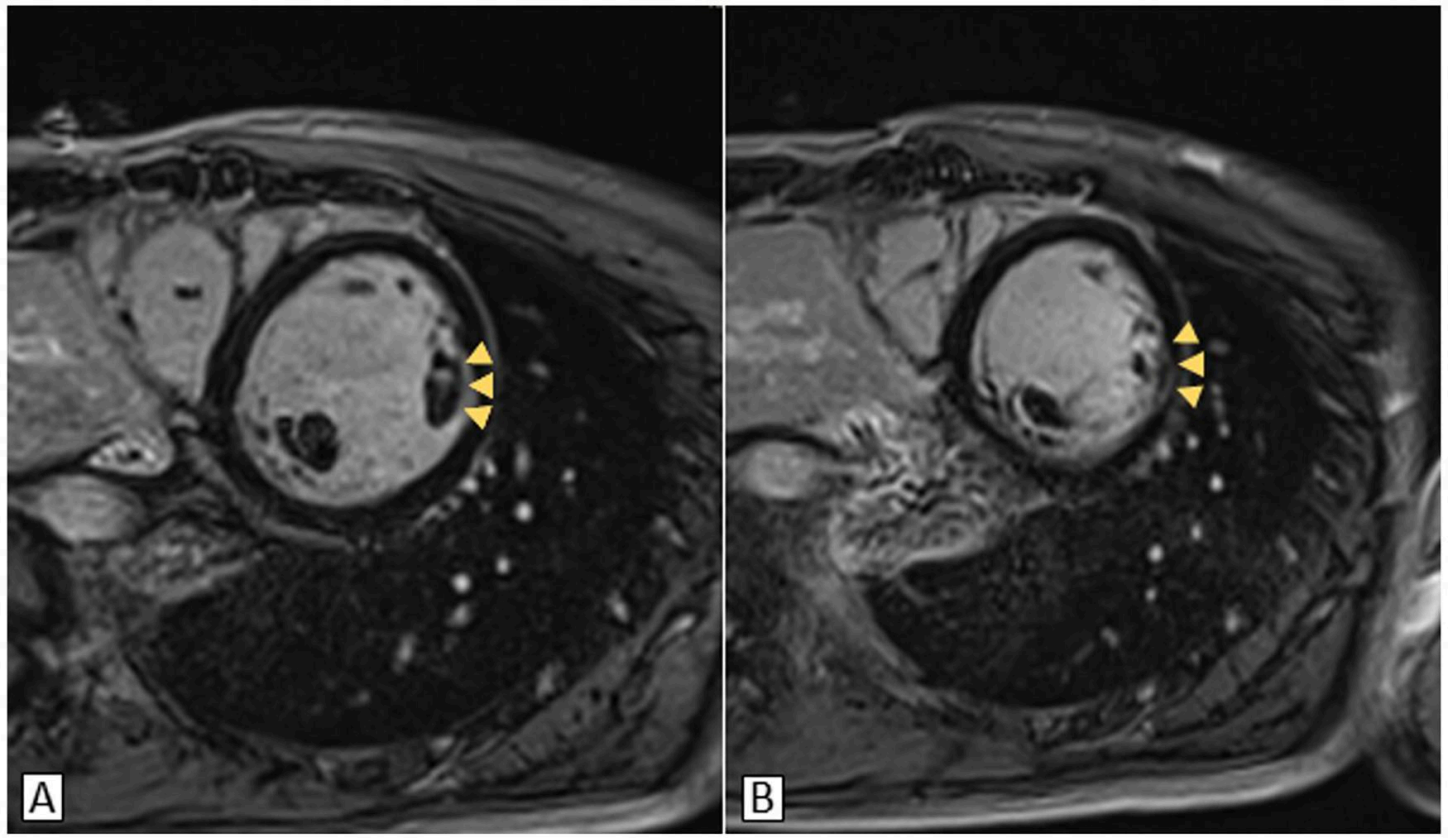
Cardiac magnetic resonance. Two consecutive mid-basal short-axis Phase Sensitive Inversion Recovery (PSIR) images **(A,B)** demonstrate intramural late gadolinium enhancement (LGE) localized in the inferolateral myocardial wall. Notably, the enhancement extends to involve the head of the anterolateral papillary muscle, reflecting fibrotic remodeling in regions critical to arrhythmogenesis in mitral valve prolapse. PSIR, phase sensitive inversion recovery; LGE, late gadolinium enhancement

Cardiac Magnetic Resonance has emerged as the leading imaging modality for evaluating MVP, uniquely combining precise functional assessment with advanced myocardial tissue characterization. Unlike CT, CMR integrates a comprehensive panel of clinically relevant information within a single imaging session. This encompasses precise quantification of mitral regurgitation severity by phase-contrast flow measurement, accurate volumetric assessment of both left and right ventricles, and detailed evaluation of myocardial tissue composition—capabilities not accessible with CT. CMR is the gold standard technique for volumetric quantification of left ventricular and atrial size, assessment of mitral regurgitation severity and comprehensive evaluation of right ventricular function - all of which are essential for identifying patients prone to adverse ventricular remodeling and arrhythmic events. Beyond chamber quantification, CMR provides unparalleled insights into myocardial composition through tissue characterization techniques such as late gadolinium enhancement (LGE), which detects focal replacement fibrosis, most commonly involving the PMs and the basal inferolateral left ventricular wall - regions that have been consistently implicated in ventricular arrhythmogenesis in MVP (Figure 4) (42). Complementary parametric mapping techniques, including native T1 mapping and extracellular volume (ECV) quantification, further increase sensitivity to diffuse interstitial fibrosis, a process that may precede the development of overt focal scarring. In parallel, T2 mapping allows identification of myocardial edema, providing additional information on potentially active or reversible myocardial injury. In addition to tissue characterization, CMR is crucial for the evaluation of dynamic valvular and annular biomechanics, including annular curling and exaggerated systolic leaflet displacement, thereby elucidating the mechanical forces transmitted to the adjacent myocardium that may promote fibrotic remodeling and arrhythmia development (43). An increased extent of MAD measured by CMR has been associated with a higher burden of VAs (13), with echocardiographic data suggesting a threshold of approximately 8.5 mm for increased arrhythmic risk (44). While LGE remains central to the identification of myocardial scar, functional CMR imaging has also demonstrated abnormal PMs dynamics, including increased peak velocity and excursion distance, further refining the understanding of PMs involvement in arrhythmic MVP. Emerging CMR techniques, such as feature-tracking and myocardial strain analysis, are increasingly being explored to standardize assessment of PMs and ventricular mechanics (45). Feature-tracking CMR (CMR-FT) allows detection of subtle abnormalities in myocardial deformation that reflect subclinical dysfunction and contribute to arrhythmia susceptibility through mechanisms including conduction heterogeneity and altered mechano-electrical coupling. Notably, reductions in global longitudinal and circumferential strain assessed by CMR-FT have been independently associated with complex VAs in patients with MVP (46). Taken together, these capabilities position CMR as the key noninvasive modality for comprehensive phenotypic and myocardial substrate characterization in selected patients with suspected arrhythmogenic MVP, while its broader prognostic integration still depends on further standardization and validation of several advanced markers. Despite these advantages, broader clinical implementation remains limited by restricted availability and resource constraints in many healthcare settings, which continue to challenge its routine incorporation into standard MVP management pathways. Currently, CMR is best viewed as the reference noninvasive modality for comprehensive anatomical and tissue characterization in selected MVP patients, particularly for ventricular function and focal fibrosis assessment by LGE. By contrast, parametric mapping and feature-tracking techniques are promising adjunctive tools that may further refine phenotyping, although their broader clinical implementation still requires greater technical standardization and prognostic validation.

### Cardiac magnetic resonance: evaluation of papillary muscle fibrosis

Papillary muscles are recognized as a key site of arrhythmogenicity in aMVP. Both autopsy series and LGE-CMR studies *in vivo* have consistently identified replacement fibrosis primarily in the basal-midventricular inferolateral wall and the papillary muscles, with a disproportionate involvement of the posteromedial papillary muscle. This pattern is thought to result from the abnormal mechanical forces associated with MVP, including superior displacement of the papillary muscles, systolic curling, and MAD (34, 47). Supporting this, Nagata et al. found myocardial fibrosis in 38% of MVP patients, predominantly localized to these regions, and independently associated with more pronounced leaflet prolapse, papillary muscle displacement, and basal systolic curling. Notably, a distinctive double-peak longitudinal strain pattern conferred additional arrhythmic risk beyond the presence of fibrosis alone (34).

Further emphasizing the clinical significance of papillary muscle abnormalities, Aquaro et al. (48) described the “Dark-Paps” sign—bilateral end-systolic hypointensity of the papillary muscles on early post-contrast cine images—in 20% of patients with ventricular arrhythmias and preserved ejection fraction. This sign strongly correlated with MVP and MAD and independently predicted major adverse cardiac events over a median follow-up exceeding six years, providing incremental prognostic information beyond both LGE and episodes of non-sustained ventricular tachycardia (48).

Despite the importance of papillary muscle fibrosis in the arrhythmic substrate of aMVP, accurately detecting it using conventional bright-blood (BB) LGE and phase-sensitive inversion recovery (PSIR) sequences is challenging. The high signal intensity of the adjacent blood pool can obscure or mimic fibrosis due to the small size and subendocardial location of the papillary muscles. Dark-blood LGE (DB-LGE) techniques address this limitation by suppressing blood signal via additional magnetization preparations such as T2 preparation, magnetization transfer, or multiple inversion pulses. This markedly improves contrast between the fibrotic myocardium and the ventricular cavity, enhancing the visibility of papillary muscle fibrosis (49).

The superiority of DB-LGE compared to BB-LGE for papillary muscle assessment in MVP has been shown prospectively by Spampinato et al., who reported detection of fibrosis in nearly 79% of patients with DB-LGE vs. only 26% with BB-LGE. Moreover, DB-LGE positivity at the posteromedial papillary muscle was uniquely correlated with biopsy-confirmed left ventricular inferobasal fibrosis (50).

Among DB-LGE methods, the Flow-Independent Dark-blood DelayEd Enhancement (FIDDLE) sequence appears particularly promising. Validated against histopathology in a canine myocardial infarction model, FIDDLE demonstrated significantly higher sensitivity and accuracy for papillary muscle scar detection than conventional delayed enhancement MRI. In clinical cohorts, FIDDLE identified papillary muscle scarring in far more cases than traditional MRI, though it remains a research tool rather than a standard clinical technique in aMVP (51). Taken together, DB-LGE approaches—including FIDDLE where available—may be considered for selected patients or within research protocols focused on papillary muscle fibrosis. However, their routine clinical incorporation in aMVP requires further prospective validation.

Beyond fibrosis extent, the spatial organization and microstructural properties of the myocardial substrate may critically influence arrhythmogenic risk. Diffusion tensor CMR offers insights into myocardial fiber orientation, microstructural integrity, and potential conduction heterogeneity, providing a mechanistic link between tissue remodeling and arrhythmic vulnerability. Nonetheless, these advanced imaging modalities have not yet been validated specifically in arrhythmic MVP and should be regarded as research tools rather than established clinical markers.

**Figure 5:**
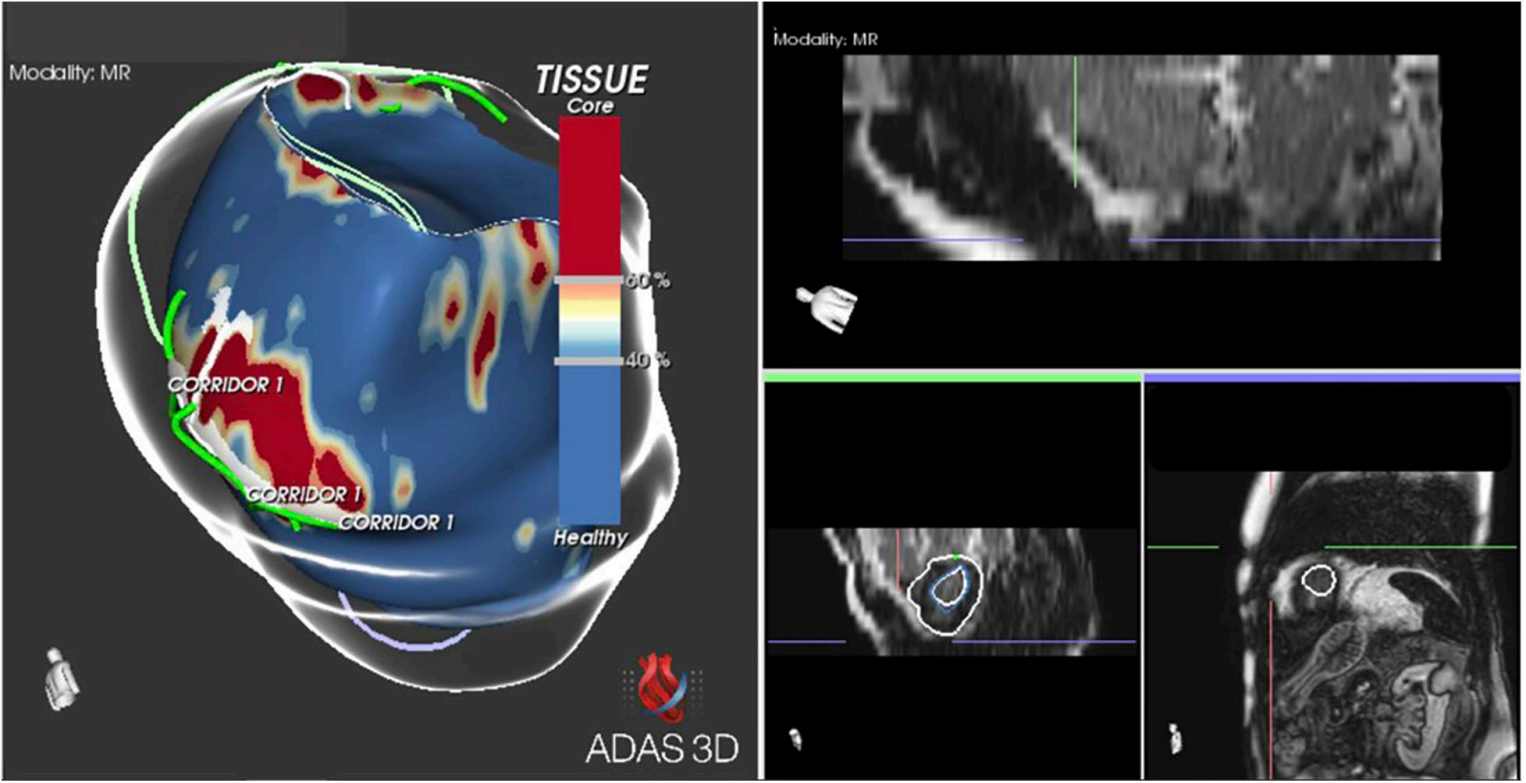
Advanced post-processing of cardiac magnetic resonance data with ADAS® 3D. Identification of conduction-relevant channels within areas of fibrosis, such as border zones and heterogeneous corridors, thanks to post-processing reconstructions. Credits to the Centre for Inherited Cardiovascular Diseases, Genomic and Precision Medicine, WellSpan Health (York, PA, USA).

In a similar vein, advanced post-processing of high-resolution LGE datasets using platforms such as ADAS® shows promise for more detailed characterization of scar architecture, including border zones and potential conducting channels (Figure 5). These features, quantified through metrics like channel burden and spatial distribution, allow a more detailed assessment of substrate complexity beyond the global fibrosis burden measured by conventional LGE. Such approaches have already been applied in ventricular tachycardia ablation and ischemic heart disease, suggesting potential utility in aMVP (52, 53). Nevertheless, their added prognostic value in this setting remains unproven, and at present, these methods should be viewed as hypothesis-generating rather than a routine clinical tool.

### Nuclear imaging and hybrid PET/MRI

**Figure 6:**
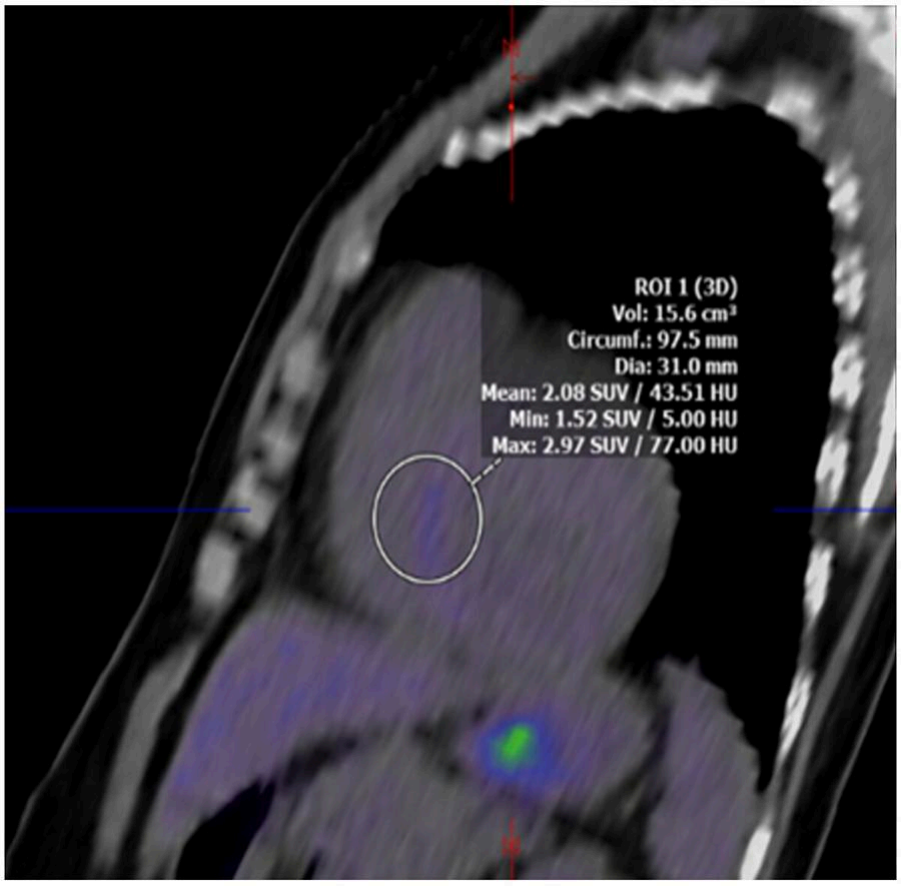
Positron emission tomography (PET) in a patient with arrhythmogenic mitral valve prolapse. The figure shows focal myocardial 18F-fluorodeoxyglucose (FDG) uptake, consistent with metabolically active myocardial involvement. The image shows a region of interest (ROI) within the basal inferolateral left ventricular wall with a volume of 15.6 cm^3^, circumference of 97.5 mm, and a diameter of 31.0 mm. Measured uptake values within the ROI include a mean standardized uptake value (SUV) of 2.08, minimum SUV of 1.52, and a maximum SUV of 2.97, supporting localized inflammatory activity in the myocardial tissue adjacent to the mitral valve apparatus. PET, positron emission tomography; FDG, fluorodeoxyglucose; ROI, region of interest.

Hybrid positron emission tomography/magnetic resonance imaging (PET/MRI) has emerged as an exploratory multimodality approach for myocardial tissue characterization in MVP, combining the anatomical and tissue characterization strengths of MRI with the ability of PET to detect metabolic alterations, most commonly through 18F-fluorodeoxyglucose (18F-FDG) uptake as a surrogate marker of possible myocardial injury (Figure 6). By extending beyond purely structural assessment, PET/MRI may provide complementary information on metabolically active myocardial involvement in selected patients with arrhythmogenic MVP, particularly when interpreted alongside CMR-based markers of fibrosis and tissue abnormality. This may be of particular interest in patients who present with ventricular arrhythmias despite the absence of extensive structural abnormalities or overt LGE-defined scar on conventional imaging. In a seminal study by Miller et al. (54), focal or focal-on-diffuse 18F-FDG myocardial uptake was frequently observed in MVP patients with mild-to-moderate mitral regurgitation and showed close spatial colocalization with regions of LGE, supporting a possible relationship between metabolically active myocardial injury and focal fibrosis. In the same setting, abnormalities in native T1, and less consistently in T2 mapping and ECV, suggested a heterogeneous pattern of myocardial tissue remodeling beyond focal scar alone. Collectively, these findings support a potential role for PET/MRI within a broader framework of myocardial tissue characterization, encompassing tissue injury, and fibrosis. At present, however, its application in arrhythmogenic MVP remains investigational, with any implications for risk assessment or therapeutic intervention considered hypothesis-generating rather than clinically validated. Consequently, PET/MRI should be regarded as an emergent research tool for mechanistic exploration in specific patients rather than a standard element of routine risk stratification protocols.

## CMR tissue characterization: From subclinical myocardial injury to fibrotic remodeling

**Figure 7:**
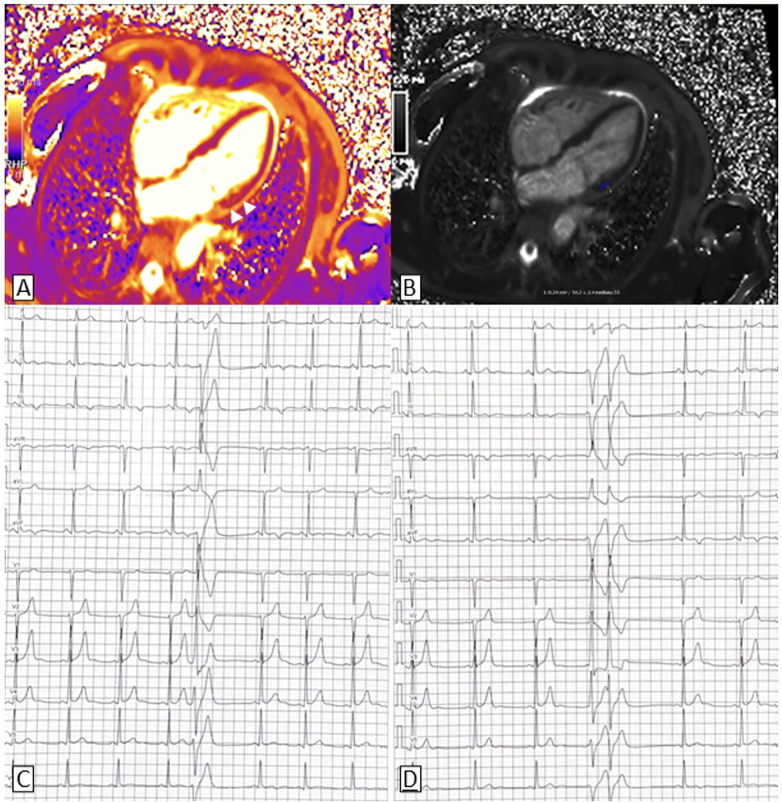
Cardiac magnetic resonance with T2 mapping technique. T2 mapping **(A,B)** in a patient with arrhythmic mitral valve prolapse (aMVP) shows focal prolongation of T2 relaxation time to 55 ms (normal range: 49 ± 5 ms) localized to the basal inferolateral wall on the diastolic four-chamber long-axis view (arrow). Holter monitoring reveals frequent single and paired premature ventricular contractions (PVCs) exhibiting a right bundle branch block morphology with superior axis **(C,D)**.

**Figure 8:**
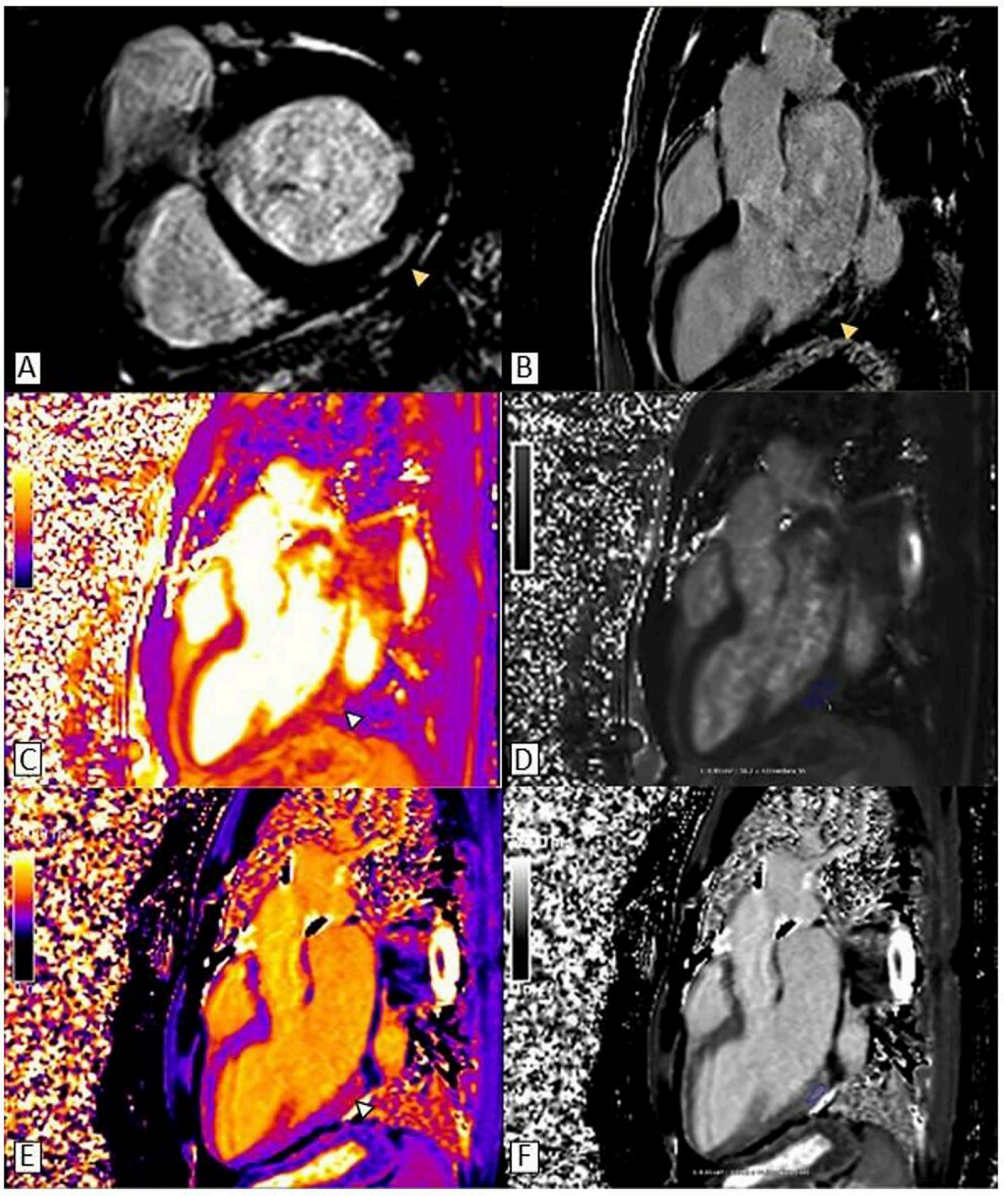
Cardiac magnetic resonance. Three–chamber PSIR image **(A)** showing basal inferolateral late gadolinium enhancement (LGE) in a young patient with arrhythmic mitral valve prolapse (aMVP). Multiparametric mapping **(B–F)** demonstrates elevated native T1 (1101 ms; normal 970 ± 70 ms) and T2 relaxation time (55 ms; normal range 49 ± 5 ms) in the basal inferolateral left ventricular segments, consistent with myocardial injury. These findings reflect subclinical myocardial tissue alterations relevant to arrhythmia risk in aMVP. Collateral finding: presence of a minimal amount of pericardial effusion. aMVP, arrhythmic mitral valve prolapse; LGE, late gadolinium enhancement; PSIR, phase sensitive inversion recovery.

In aMVP, CMR-based tissue characterization may be conceptualized as a continuum ranging from potentially active or reversible myocardial injury, mainly assessed by T2 mapping, to diffuse interstitial fibrotic remodeling, assessed by native T1 mapping and extracellular volume quantification, and finally to established focal replacement fibrosis detected by LGE. Recent studies have increasingly focused on the presence of myocardial injury in aMVP through advanced imaging techniques, primarily CMR using native T2 mapping and hybrid PET/MRI (54, 55). Native T2 mapping, a sensitive and reliable marker of myocardial injury, consistently demonstrates localized myocardial tissue changes predominantly within the basal inferolateral segments of the left ventricle in patients with MVP (55). Figure 7 illustrates this pattern by showing focal prolongation of T2 relaxation time within the basal inferolateral wall in a patient with aMVP This myocardial damage often precedes the development of fibrosis detectable by LGE, indicating a potentially reversible early stage before irreversible fibrotic remodeling ensues (56, 57). In this context, hybrid PET/MRI techniques assessing 18F-fluorodeoxyglucose (18F-FDG) uptake offer complementary evidence of ongoing myocardial injury, with FDG uptake frequently colocalizing to areas of tissue abnormalities and fibrosis identified on CMR—even when overt structural changes are not evident by LGE (58, 59). These findings collectively support a possible early role of myocardial tissue damage within the pathophysiological cascade linking mechanical myocardial stress to myocardial remodeling and arrhythmogenesis in MVP. Patient cohorts examined tend to include individuals both with and without mitral annular disjunction (MAD), a condition associated with an increased arrhythmic burden and more pronounced imaging abnormalities (60). Elevated native T2 and T1 mapping values correlate strongly with higher prevalence of ventricular arrhythmias, supporting the hypothesis that myocardial injury, tissue alterations, and arrhythmogenic substrate development are interrelated processes (53, 61). Further, extracellular volume (ECV) quantification elucidates diffuse interstitial fibrosis extending beyond focal LGE-detected regions, particularly adjacent to sites of annular disjunction (62). Figure 8 provides an example of this multiparametric approach, demonstrating the coexistence of basal inferolateral LGE with elevated native T1 and T2 values in a patient with aMVP.

Several important limitations should be acknowledged when interpreting these findings. Most available studies have been conducted in relatively small and selected cohorts, often enriched for arrhythmic phenotype or referral to advanced imaging centers. In addition, T1/T2 mapping results may be influenced by scanner type, field strength, acquisition protocol, and center-specific reference values, and universally accepted thresholds for clinical decision-making are not yet available. Finally, the temporal relationship between myocardial injury, tissue remodeling, fibrosis, and ventricular arrhythmias remains biologically plausible but has not yet been fully established in large longitudinal studies. Importantly, sex-based differences have been observed, with female patients showing a stronger association between interstitial fibrosis, assessed by ECV, and arrhythmic phenotype (61). In the study by Tastet et al., this association was particularly evident in women with AMVP, including a more diffuse pattern of ECV expansion not limited to the inferolateral segments typically exposed to abnormal mechanical traction (61). These findings do not currently support female sex as an isolated indication for advanced CMR referral or intensified follow-up, but they suggest that sex may act as a clinically relevant risk modifier. Therefore, in women with MVP, especially when ventricular arrhythmias, unexplained syncope, high-risk morphological features, MAD, LGE, or abnormal mapping parameters are present, a lower threshold for comprehensive CMR tissue characterization and closer rhythm/imaging surveillance may be reasonable, while awaiting prospective validation. The synthesis of these multiparametric imaging biomarkers, in combination with functional and clinical data, enables a comprehensive assessment of myocardial remodeling in MVP, highlighting subclinical myocardial damage and early tissue alterations as potentially initial features of myocardial remodeling that precede fibrotic transformation and electrical instability. Taken together, these findings support an evolving model in which myocardial injury, tissue remodeling, and fibrosis may represent interconnected stages of myocardial remodeling in aMVP, although the temporal sequence and clinical implications of this progression remain incompletely defined. An overview of key studies supporting these concepts is summarized in Table 1.

**Table 1:** Key studies investigating subclinical myocardial tissue changes—including metabolically active injury, fibrosis, and remodeling—and their link to arrhythmic risk in mitral valve prolapse (MVP) using advanced imaging.

Study (Author, Year)	Population	Imaging technique	Sample size (n)	Main findings
Tung et al. 2015 (58)	Patients with unexplained cardiomyopathy and arrhythmias	PET (18F-FDG)	78	Occult metabolically active myocardial injury; underscores the contribution of myocardial injury in arrhythmogenesis
Miller et al. 2020 (59)	Degenerative MVP with ventricular ectopy	Hybrid PET/MRI (18F-FDG)	14	Focal myocardial FDG uptake reflecting metabolically active tissue abnormalities; correlates with fibrosis and myocardial impairment
Pavon et al. 2021 (60)	MVP patients with MAD	CMR extracellular volume (ECV) quantification	30	Increased extracellular volume (ECV) adjacent to annular disjunction regions; diffuse fibrosis associated with arrhythmic phenotypes
Guglielmo et al. 2023 (46)	Arrhythmic MVP patients vs. controls	CMR native T1 and T2 mapping	52	Elevated native T1 and T2 values linked to arrhythmic risk; early myocardial tissue alterations identified prior to fibrotic remodeling
Cecere et al. 2023 (62)	Arrhythmic MVP patients	LGE quantification + ECV	66	LGE detected in 62% of patients; fibrosis ∼2.4% of LV mass; elevated ECV observed near fibrotic areas
Mangini et al. 2025 (55)	MVP patients with and without MAD	CMR T2 mapping	39	Raised T2 values in the basal inferolateral left ventricular segments; associated with arrhythmic burden and subclinical tissue changes
Cristin et al. 2025 (25)	Arrhythmic MVP patients	Multimodality Imaging (CMR, PET, echocardiography)	75	Multimodality imaging improves arrhythmic risk stratification by integrating metabolically active myocardial damage and fibrosis data
Tastet et al. 2024 (61)	MVP patients, sex-based differences	CMR T1 mapping and ECV	65	Females exhibit higher ECV and fibrosis burden; correlated with increased risk of malignant arrhythmias

These studies include different MVP populations assessed primarily with cardiac magnetic resonance techniques (T1/T2 mapping, ECV, LGE) and PET/MRI. Results emphasize subclinical myocardial tissue changes and mechanical abnormalities associated with higher arrhythmic burden, highlighting the value of multiparametric imaging biomarkers for risk stratification and clinical management in MVP. LV, left ventricle; MAD, mitral annular disjunction; aMVP, arrhythmic mitral valve prolapse; CMR, cardiac magnetic resonance; LGE, late gadolinium enhancement; ECV, extracellular volume.

### Late gadolinium enhancement

**Figure 9:**
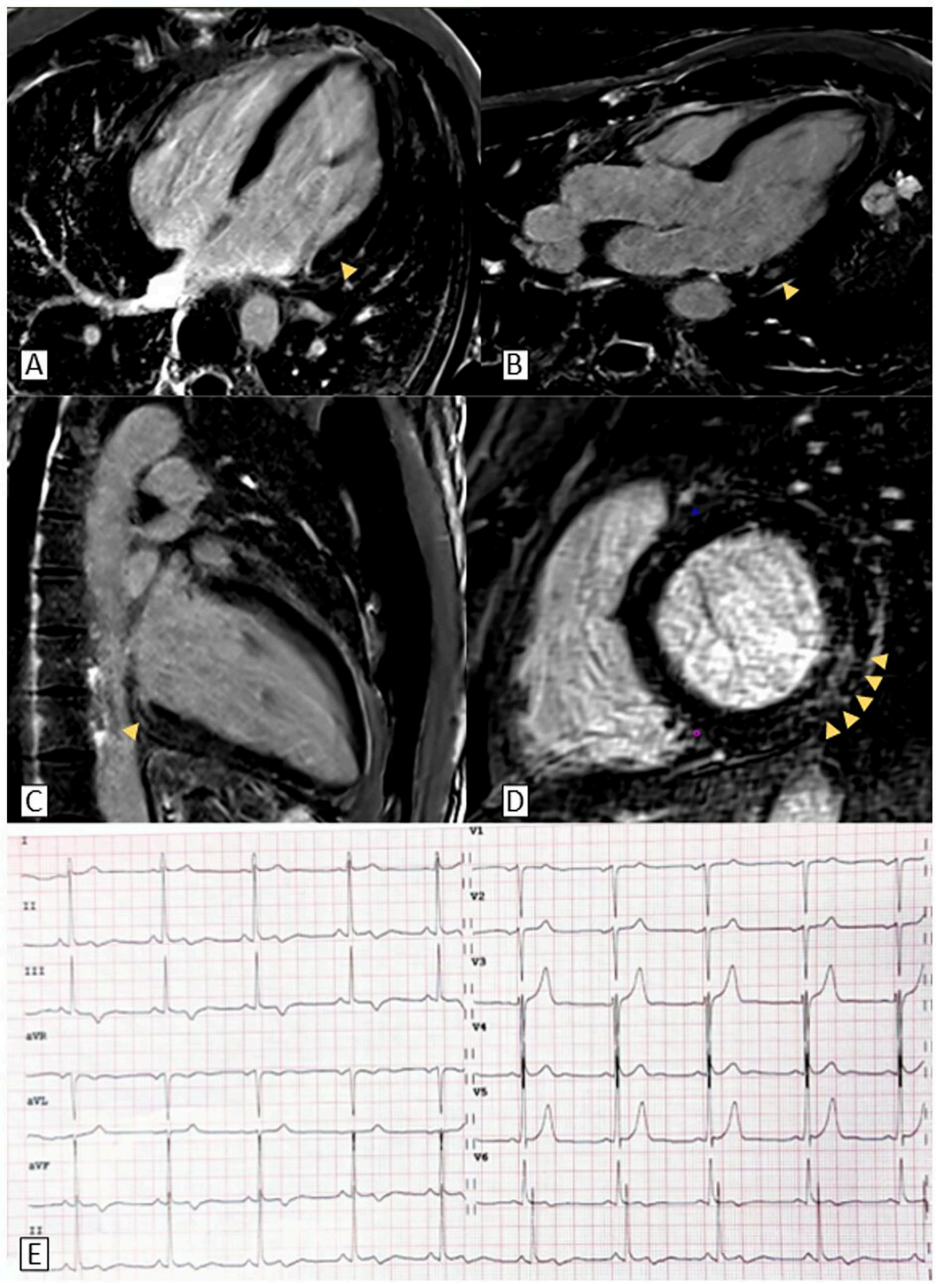
Cardiac magnetic resonance (CMR). CMR late gadolinium enhancement (LGE) in arrhythmic MVP shows subepicardial-intramural enhancement mainly in the inferolateral wall across standard long- and short-axis PSIR views **(A–D)**. Blue and pink dots mark myocardial regions for analysis. Electrocardiographic features **(E)** include characteristic T wave inversion or biphasic T waves observed in the inferior leads in the inferior leads (II, III, aVF) and lateral (V6) lead. ECG: CMR, cardiac magnetic resonance; LGE, late gadolinium enhancement; PSIR, phase sensitive inversion recovery.

Late Gadolinium Enhancement plays a central role in the detection of myocardial fibrosis in MVP, and remains one of the most relevant CMR markers for identifying focal replacement scar within the arrhythmogenic substrate (Figure 9). In MVP, fibrotic involvement is most frequently localized to the basal inferolateral left ventricular wall and the PMs insertion sites, regions that have been consistently implicated in arrhythmia initiation and maintenance (6). Within a broader multimodality and clinical assessment, LGE therefore provides important information on the presence and distribution of established myocardial injury. Although advanced parametric mapping techniques may identify earlier and more diffuse myocardial abnormalities, the presence of replacement fibrosis detectable by LGE remains the imaging finding most consistently associated with ventricular arrhythmia burden and adverse phenotypic expression in MVP. However, variability in LGE acquisition and post-processing methodologies has historically limited the consistency and clinical interpretability of fibrosis assessment. Accumulating evidence supports the use of a 5–standard deviation (SD) threshold as the most robust and conservative approach for LGE quantification in MVP, yielding reproducible fibrosis estimates typically in the range of 2%–3% of total left ventricular mass (62). In contrast, lower thresholds, such as 2- or 3-SD, tend to substantially overestimate fibrotic burden and may reduce the specificity and prognostic value of LGE findings. Adoption of standardized LGE quantification strategies therefore enhances both the reliability and clinical utility of fibrosis detection, facilitating more accurate identification of patients at heightened arrhythmic risk. Nonetheless, it is important to acknowledge that a subset of patients with malignant VAs may exhibit only limited or even absent LGE, underscoring that myocardial fibrosis represents only one component of a complex and heterogeneous arrhythmogenic substrate. Overall, LGE remains a key maging tool for characterizing focal myocardial fibrosis in MVP and for contributing to a more refined arrhythmic risk assessment when interpreted together with quantitative tissue characterization, functional imaging markers, and clinical data.

### Pathophysiology and clinical interpretations

The imaging findings discussed above are consistent with an evolving pathophysiological model in which repetitive mechanical stress, myocardial injury and fibrotic remodeling may contribute jointly to arrhythmogenesis in MVP. Rather than repeating the individual imaging observations, this section focuses on how such findings may be interpreted within a broader mechanistic framework. In this context, myocardial injury detected by advanced imaging methods such as T2 mapping may represent an early manifestation of myocardial remodeling related to abnormal leaflet motion, leaflet redundancy, and mitral annular disjunction (4, 5). PET-based observations further support the possibility that myocyte injury may coexist with, or contribute to, myocardial remodeling in selected patients, although FDG uptake remains a nonspecific marker and should be interpreted with caution (58, 61). Taken together, these findings support a plausible link between mechanical stress, tissue injury, and subsequent fibrotic remodeling, but the temporal sequence and causal hierarchy of these processes remain incompletely defined. The pathophysiological framework linking mechanical stress, myocardial injury and increased ECV, fibrosis, and ventricular arrhythmias in MVP is increasingly supported by multimodality imaging, but it should still be regarded as an evolving model rather than a fully validated sequence applicable to all phenotypic subsets. This distinction is important when translating mechanistic observations into clinical interpretation and mechanical factors alone are unlikely to explain the full heterogeneity of arrhythmic MVP. Myocardial injury may not solely be a consequence of local biomechanical stress but could also reflect an intrinsic myocardial vulnerability influenced by genetic predisposition and other modifiers. This is supported by findings of increased ECV in regions of the myocardium distant from mechanical stress sites, as well as the presence of pathogenic mutations in cardiomyopathy-associated genes among patients with MVP (63, 66, 68). More broadly, these observations suggest that genetic susceptibility and biomechanical stress may act in concert, helping to explain why severe mechanical abnormalities are not uniformly associated with arrhythmias, whereas some patients with less marked valvular changes still develop malignant ventricular arrhythmias or SCD (69, 70). At present, this framework is useful for interpreting multimodality imaging findings, but its temporal sequence and direct implications for management remain to be established. Therefore, current imaging findings should be interpreted primarily as markers associated with arrhythmic phenotype and myocardial remodeling rather than as definitive proof of a linear causal sequence or as stand-alone determinants of management.

### Genetic susceptibility and phenotypic heterogeneity

Familial clustering of MVP has long been recognized, and both Mendelian and polygenic contributions appear to participate in disease susceptibility, distinct from syndromic prolapse associated with heritable connective tissue disorders. Genome-wide association studies have identified common susceptibility loci, including genes involved in valvular development and structural integrity such as LMCD1 and TNS1 (63), while more recent meta-analytic data have expanded the polygenic architecture of MVP to include pathways related to TGF-β signaling, cytoskeletal organization, and cardiomyopathic remodeling (64, 65). These observations support the concept that, in at least some patients, the substrate for MVP extends beyond leaflet morphology alone and may reflect a broader predisposition involving developmental and myocardial factors. At the same time, rare pathogenic variants in genes such as FLNC, LMNA (66, 67), SCN5A (68), and HCN4 (69) have been reported in individual patients or families with arrhythmic MVP and malignant ventricular arrhythmias, raising the possibility that, in selected cases, the arrhythmic phenotype may overlap with an underlying cardiomyopathic or channelopathy susceptibility (70). Variants in ion-channel genes such as SCN5A and HCN4 may contribute to altered cardiac excitability and conduction properties, thereby promoting electrical instability independently of, or in addition to, structural abnormalities. For instance, SCN5A encodes the cardiac sodium channel Nav1.5, which plays a central role in action potential initiation and impulse propagation; pathogenic variants have been associated with conduction slowing, repolarization abnormalities, and ventricular arrhythmias (68). Similarly, variants in genes associated with structural cardiomyopathies, such as FLNC and LMNA, may predispose to myocardial fibrosis, cytoskeletal disorganization, and altered mechano-electrical coupling. These processes may create a vulnerable substrate characterized by conduction heterogeneity and increased arrhythmic susceptibility, particularly in regions subjected to repetitive mechanical stress, such as the basal inferolateral wall and papillary muscles. Recent evidence suggests that these genetic factors may act as effect modifiers rather than primary causes, supporting a “two-hit” model in which intrinsic myocardial vulnerability interacts with valvular mechanical stress to promote fibrosis and arrhythmogenesis (70). This may help explain why severe mechanical abnormalities are not uniformly associated with arrhythmias, whereas some patients with less marked leaflet changes still develop malignant ventricular arrhythmias or sudden cardiac death. Although genetic testing is not currently incorporated into routine clinical risk stratification for arrhythmogenic MVP (8), genetic susceptibility may be viewed as a plausible modifier of myocardial vulnerability, acting in concert with leaflet prolapse, annular dynamics, and tissue remodeling rather than as an alternative explanation to them. From a practical perspective, this reinforces the need to interpret imaging findings within a broader phenotypic framework that allows for heterogeneous underlying susceptibility. Practical clinical interpretation of imaging findings. In clinical practice, multimodality imaging in arrhythmogenic MVP should be interpreted as part of an integrated framework that includes symptoms, ECG abnormalities, ventricular ectopy burden, and mitral valve phenotype, rather than as a stand-alone determinant of management. Echocardiography remains the first-line modality for identifying MVP morphology, mitral regurgitation severity, and dynamic mechanical features such as annular abnormalities. CMR is currently the most clinically relevant second-line modality when an arrhythmogenic phenotype is suspected, particularly in patients with unexplained syncope, documented non-sustained ventricular tachycardia, complex ventricular ectopy, or other phenotypic high-risk features, because it enables assessment of ventricular remodeling, MAD, and focal myocardial fibrosis. By contrast, advanced tissue characterization with mapping techniques may refine phenotyping but still requires broader standardization and validation. PET/MRI currently remains investigational and may be considered only in highly selected cases or expert centers when additional mechanistic insight into myocardial injury is sought. Importantly, imaging findings should be viewed as contributors to comprehensive arrhythmic evaluation and follow-up intensity, rather than as triggers in isolation for ICD implantation or other major therapeutic decisions (Table 2).

**Table 2:** Integration of multimodality imaging findings for risk stratification in suspected arrhythmogenic mitral valve prolapse.

Imaging modality/finding	What it may indicate	Potential clinical consequence	Current maturity of evidence	Important caveat
Echocardiographic MVP phenotype with severe myxomatous degeneration, bileaflet prolapse, marked leaflet redundancy, or MAD	Higher-risk structural phenotype	Supports closer rhythm surveillance and consideration of further phenotypic assessment	Moderate	Should not be used in isolation for arrhythmic risk stratification
Significant mitral regurgitation and/or LV remodeling	Hemodynamic burden and possible excess risk context	Relevant for valve management and overall risk assessment	High	Arrhythmic risk is not determined by mitral regurgitation severity alone
CMR-detected LGE in papillary muscles or inferolateral/basal LV wall	Focal replacement fibrosis associated with higher arrhythmic risk phenotype	Supports more comprehensive arrhythmic work-up and follow-up intensification	Moderate	Prognostic thresholds and management guidelines remain to be standardized
CMR evidence of MAD extension, LV enlargement, or systolic dysfunction	Structural remodeling and adverse phenotype enrichment	Strengthens the indication for integrated imaging–rhythm evaluation	Moderate	Association does not mandate direct treatment decisions
Abnormal T1/T2 mapping or elevated ECV	Possible diffuse fibrosis and/or myocardial tissue alterations	May refine phenotyping in selected patients	Low to moderate	Variability due to center, scanner, and protocol limit widespread clinical use
PET/MRI demonstrating focal metabolically active myocardial involvement	Possible metabolically active myocardial involvement	May provide mechanistic insight in selected expert-center cases	Low/investigational	Not a routine clinical decision-making tool currently
Combination of high-risk imaging phenotype plus syncope, complex ventricular arrhythmias (VA), or nonsustained ventricular tachycardia (NSVT)	Higher suspicion of clinically relevant arrhythmic MVP	Justifies intensified rhythm monitoring and specialist arrhythmia evaluation	Moderate	Implantable cardioverter-defibrillator (ICD) decisions remain individualized and should not rely solely on imaging

Summary of key multimodal imaging findings in suspected arrhythmogenic mitral valve prolapse, their clinical significance, evidence strength, and important limitations to guide risk stratification and management.

## Discussion

A comprehensive imaging strategy that integrates the complementary strengths of echocardiography, CT, CMR, and hybrid PET/MRI enables a nuanced and multidimensional assessment of mitral valve morphology, myocardial integrity, and tissue-level remodeling. Such an approach is increasingly recognized as essential for accurate risk stratification in patients with arrhythmic MVP (71, 72). Overall, the reviewed literature is consistent with a possible continuum from early myocardial injury to more established fibrotic remodeling, although this progression remains incompletely validated across different MVP phenotypes. In parallel, strain analysis derived from CMR may provide additional phenotypic information by detecting subtle myocardial dysfunction that may not be apparent on purely structural imaging.

Current limitations of the evidence should also be acknowledged. The available literature on multimodality imaging in arrhythmic MVP remains heterogeneous in design, sample size, referral setting, and endpoint definition. Many studies are retrospective, single-center, and enriched for patients with ventricular arrhythmias or advanced phenotypic features, which may limit generalizability to the broader MVP population. In addition, imaging protocols and post-processing approaches, particularly for LGE quantification and parametric mapping, are not fully standardized across centers. These limitations should be considered when interpreting the apparent consistency of imaging associations across studies. As a result, current evidence is stronger for phenotypic association and mechanistic plausibility than for immediate broad implementation across unselected MVP populations. Large, prospective studies are needed to establish standardized imaging criteria, validate robust prognostic biomarkers, and clarify the temporal interplay between myocardial injury, fibrosis, and VAs. In parallel, interventional trials targeting myocardial injury and fibrotic pathways are eagerly awaited, as they hold the potential to translate these mechanistic insights into meaningful advances in the prevention and management of life-threatening arrhythmic complications in MVP.

## Conclusions

Multimodality imaging increasingly supports the presence of early myocardial remodeling in selected patients with arrhythmic MVP, including markers of myocyte injury and fibrosis. At present, the main contribution of these findings lies in improved phenotypic characterization and in the generation of pathophysiological and prognostic hypotheses, while their precise role in clinical decision-making still requires further validation. Complementary imaging modalities, including CMR and PET, further refine risk evaluation by revealing myocardial fibrosis and injury that may remain undetected by conventional echocardiography. Further prospective multicenter studies will be essential to define standardized thresholds, validate incremental prognostic value, and clarify how these biomarkers should be integrated into clinical pathways. Ultimately, the development of robust multiparametric risk models, combining clinical variables, advanced imaging biomarkers, and electrophysiological data will be critical to enabling truly personalized management strategies for patients at highest arrhythmic risk.
